# A Framework for Real-Time Gestural Recognition and Augmented Reality for Industrial Applications

**DOI:** 10.3390/s24082407

**Published:** 2024-04-10

**Authors:** Winnie Torres, Lilian Santos, Gustavo Melo, Andressa Oliveira, Pedro Nascimento, Geovane Carvalho, Tácito Neves, Allan Martins, Ícaro Araújo

**Affiliations:** 1 Electrical Engineering Department, Center of Technology, Federal University of Rio Grande do Norte—UFRN, Natal 59072-970, Brazil; gustavocosta@ic.ufal.br (G.M.); andressa@ic.ufal.br (A.O.); allan@dee.ufrn.br (A.M.); 2 Computing Institute, A. C. Simões Campus, Federal University of Alagoas—UFAL, Maceió 57072-970, Brazil; lgps@ic.ufal.br (L.S.); phbn@ic.ufal.br (P.N.); glcf@ic.ufal.br (G.C.); icaro@ic.ufal.br (Í.A.); 3 Department of Exact Sciences, Center for Applied Sciences and Education, Federal University of Paraíba, Rio Tinto 58297-000, Brazil; tacito@dcx.ufpb.br

**Keywords:** augmented reality, gesture recognition, web application, A-Frame

## Abstract

In recent decades, technological advancements have transformed the industry, highlighting the efficiency of automation and safety. The integration of augmented reality (AR) and gesture recognition has emerged as an innovative approach to create interactive environments for industrial equipment. Gesture recognition enhances AR applications by allowing intuitive interactions. This study presents a web-based architecture for the integration of AR and gesture recognition, designed to interact with industrial equipment. Emphasizing hardware-agnostic compatibility, the proposed structure offers an intuitive interaction with equipment control systems through natural gestures. Experimental validation, conducted using Google Glass, demonstrated the practical viability and potential of this approach in industrial operations. The development focused on optimizing the system’s software and implementing techniques such as normalization, clamping, conversion, and filtering to achieve accurate and reliable gesture recognition under different usage conditions. The proposed approach promotes safer and more efficient industrial operations, contributing to research in AR and gesture recognition. Future work will include improving the gesture recognition accuracy, exploring alternative gestures, and expanding the platform integration to improve the user experience.

## 1. Introduction

Technological evolution in recent decades has drastically transformed the industrial landscape, promoting automation, efficiency, and safety. Within this context, the convergence of emerging technologies, such as augmented reality (AR) and gesture recognition, has emerged as an innovative and highly promising approach. This fusion enables the creation of interactive and immersive environments that provide practical solutions for displaying and controlling industrial equipment parameters, revolutionizing modern industry operations.

Gesture recognition plays an important role in AR applications. It enables users to interact with virtual objects and enhances the overall user experience. Several papers discuss the use of hand gesture recognition in AR. Ref. [1] develops a software-based framework for hand gesture recognition on smartphones, enabling AR experiences comparable to head-mounted displays. Ref. [2] provides a comprehensive overview of augmented reality applications in smart facility management, offering valuable information and analysis for this evolving field.

Ref. [3] presents an algorithm for dynamic gesture recognition and prediction in AR-assisted assembly training, which evaluates hand operations in real time. Ref. [4] introduces a dataset and a recalibration technique for electromyography-based gesture recognition in AR, bridging the gap between offline and online datasets. Ref. [5] proposes a framework for hand gesture recognition on smartphones to improve the overall user experience in smartphone-based AR applications. Ref. [6] explores how a hand gesture recognition interface based on MediaPipe enhances interaction in virtual and augmented reality (VR/AR), contributing to a more immersive and intuitive experience in VR/AR environments, providing advantages in interacting with virtual objects.

According to [7], augmented reality, by superimposing virtual information onto the real world, provides an intuitive and contextualized interface for industrial operators, significantly improving operational efficiency and precision. Furthermore, recent research, such as the study [8], demonstrates that gesture recognition offers a natural and practical approach to interacting with control systems, allowing for the manipulation of equipment parameters with simple and intuitive gestures.

In this article, we present a web architecture and a practical application of augmented reality with gesture recognition that focuses on controlling the parameters of industrial equipment. The significance of this research lies in its direct applicability to industrial operations, which increasingly demand efficiency, safety, and precision. It is important to note that the proposed system can utilize a wide range of industrial controllers, provided that they possess appropriate communication protocols.

Furthermore, the proposed architecture is hardware-agnostic regarding augmented reality, enabling its implementation on various devices, from smartphones and tablets to equipment specifically designed for augmented reality. The architecture proposed in this article enables the use of augmented reality on lower-cost equipment, making the use of these tools more accessible. In this context, augmented reality delivers contextual information and visual assistance directly into the operators’ field of view. At the same time, gesture recognition empowers a more natural and intuitive interaction with equipment control systems. The architecture proposed in this article is demonstrated on an experimental bench.

## 2. Augmented Reality

Augmented reality has been a part of the technological landscape since the 1960s when Ivan Sutherland pioneered the development of the first head-mounted display system [9]. However, it was not until 1992 that Caudell and Mizzel proposed the term “AR” by defining it as a technology that enhances the visual field of the user with task-relevant information, distinguishing it from VR [10]. AR, which overlays graphic objects on the user’s real-world view, has become a popular tool across various industries and a key component of Industry 4.0 [11].

Industry 4.0 integrates cyber–physical systems into manufacturing processes, using data from sensors and actuators [12]. It aims to promote customized, information-driven, and digital services, creating new business models while improving productivity, product quality, and efficiency [13,14].

Augmented reality within Industry 4.0 has seen cross-disciplinary applications, such as supporting education, industrial maintenance, and controlling industrial robots [15].

In mobile development for AR applications, options such as React Native and Flutter offer the ability to create native apps with AR features. Languages like Kotlin/Java and Swift/Objective-C remain robust choices for AR development, while cross-platform frameworks like Xamarin, Ionic, and NativeScript ensure consistent AR experiences across multiple platforms. In intermediate server and control programming, Node.js, Express.js, Django, Ruby on Rails, Go, and ASP.NET provide various options for building efficient and scalable applications, depending on project requirements and developer preferences.

In all of the applications mentioned above, augmented reality is used to show digital information in real time alongside visual information in the real world. However, user interaction with the virtual world may also be necessary. An alternative to this interaction is the use of gesture recognition.

## 3. Gesture Recognition

Gesture recognition uses a set of techniques that allow computers to recognize human gestures [16,17,18]. It has many potential applications, such as device control [19,20], interaction with virtual environments [21], and the real-time translation of sign language [22,23,24].

A traditional gesture recognition pipeline can be divided into the following steps: hand image acquisition, hand segmentation, feature extraction, and gesture recognition. Many challenges are found in implementing this pipeline, e.g., occlusion of the hands. However, the great diversity of human gestures, varying from person to person, can be considered one of the greatest.

Using frameworks for gesture recognition is common and seeks to simplify development, improving accuracy and reliability. They usually include the necessary tools to carry out the pipeline steps presented previously. OpenPose is a framework for estimating poses in real time [25]. Similarly, the Google-developed MediaPipe [26] is a popular framework for computer vision and machine learning.

In the augmented reality scenario, gesture recognition can be a key technology, allowing interaction with AR content [27,28]. Gesture recognition is used in AR to control AR content, such as moving or rotating objects, interacting with AR menus, and navigating AR environments. There are many different approaches to gesture recognition for AR. Some of the most common approaches include image-based gesture recognition, depth-based gesture recognition, which uses depth sensors to track the user’s hand movements and identify gestures, and IMU-based systems.

A distinction between two essential types of gesture is described in [29]: static and dynamic gestures. A gesture detected only by recognizing a specific hand shape is considered static. It can be invariant to rotation or tied to a particular orientation of the hand. Temporal and spatial information, such as the path of the hand, is not considered in recognizing this type of gesture [30]. A dynamic gesture uses spatial information, such as the path of the hand or individual joints. This type of gesture is recognized by detecting movement patterns. Thus, the previously defined behavior can be recognized and used [30].

**Figure 1:**
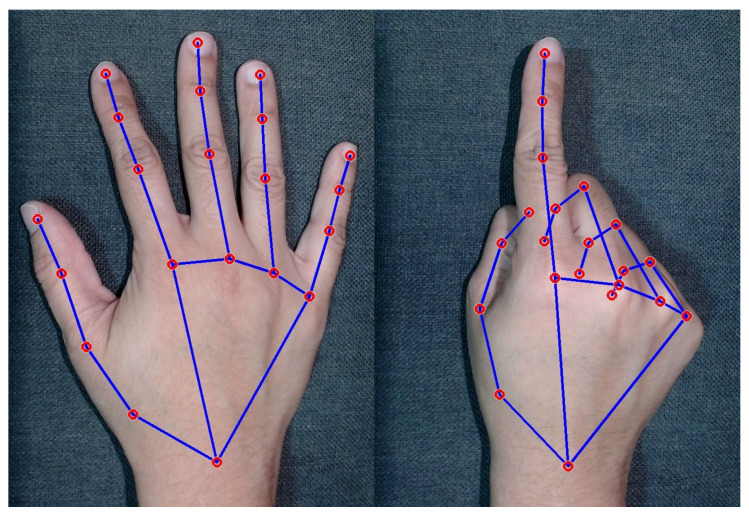
Static gesture.

**Figure 2:**
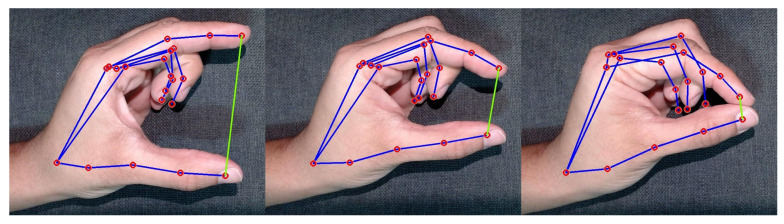
Dynamic gesture.

A typical static gesture would be the “index finger raised or open hand”, as shown in Figure 1, while realizing a tweezers movement created from the thumb and index finger would be considered a dynamic gesture, as shown in Figure 2. Performing a pistol gesture by simply moving your thumb down is a dynamic gesture with a single finger movement [30].

To enhance the understanding of this study, it should be noted that Figure 1 and Figure 2, illustrating a static gesture and a dynamic gesture, respectively, were meticulously designed to represent the signals used in our research accurately. These figures go beyond mere illustration, directly mirroring the types of gestures our system is adept at recognizing and processing. The precision in these depictions is essential, as they precisely outline how the algorithm interprets each movement, offering a clear insight into the system’s capability to distinguish and classify specific gestures. This level of clarity is crucial, not only for the integrity of experimental tests but also for the practical implementation of the system in real-world scenarios, where the accuracy in gesture recognition can profoundly influence the usability and efficiency of user–system interactions.

## 4. System Description

**Figure 3:**
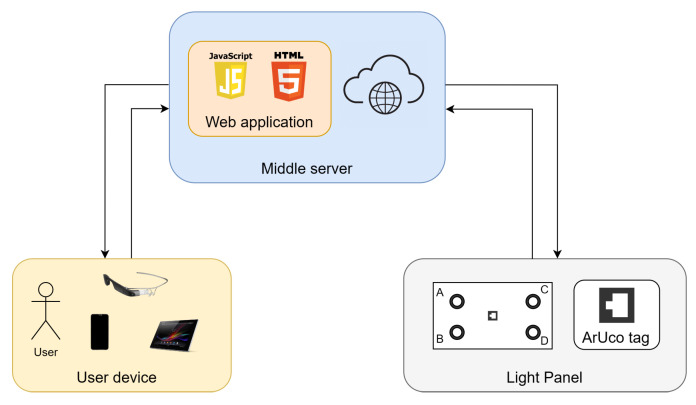
Proposed system architecture.

The proposed system architecture is illustrated in Figure 3, with its implementation consisting of four main components, which will be detailed in the following subsections: the user device, the web application, the middle server, and the light panel as the chosen end system.

The web application is accessible through a browser on a user’s device equipped with a camera. When the camera is directed toward the light panel, the system identifies the board using an ArUco tag.

In the context of augmented reality and gesture recognition, ArUco tags are essential as markers that facilitate accurate and efficient object tracking in real time. These square markers, identifiable by their unique binary patterns, allow for the seamless overlay of digital information onto the physical world, enhancing interaction within AR applications. The use of ArUco tags, as developed by [31], provides a robust method to recognize and position AR content, which is crucial to merging virtual and real environments, offering a tangible interface for users to interact with digital elements through natural gestures.

Subsequently, the application activates its AR elements and initiates hand recognition, allowing user interaction. Users can pre-configure gestures linked to specific actions to select individual lights or collectively control them, adjusting their intensity values; the middle server orchestrates this entire process.

### 4.1. User Device

Gesture recognition in augmented reality applications is crucial to enhancing user interaction with the virtual environment. To this end, a variety of user devices can be successfully employed. Tablets and smartphones are two prominent examples of user devices that have excelled in this scenario. Equipped with advanced sensors, such as accelerometers, gyroscopes, and high-resolution cameras, these devices can effectively track the movements of the user’s hand and body, enabling a more immersive and intuitive augmented reality experience.

**Figure 4:**
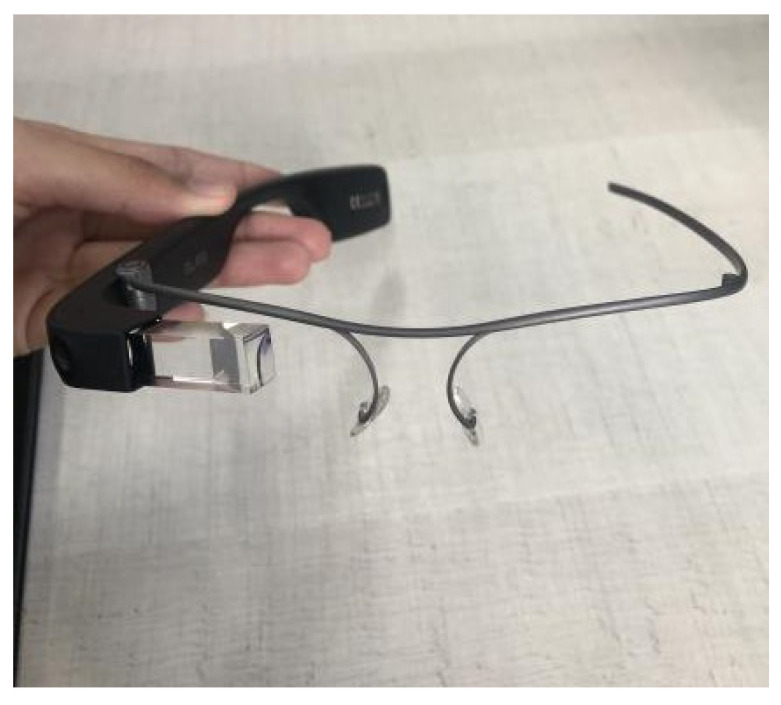
Google Glass Enterprise Edition 2 device.

Furthermore, head-mounted augmented reality devices, such as Google Glass and Hololens, also significantly implement gesture recognition. Equipped with cameras and proximity sensors, these glasses can detect subtle gestures made by the user in real time. This allows for interaction without the need to touch a screen or use external devices, providing a truly hands-free and integrated AR experience. Therefore, the combination of these devices, such as tablets, smartphones, and glasses, offers a diverse range of options for gesture recognition in augmented reality applications, catering to different user needs and preferences. When conducting the tests for this study, we utilized the Google Glass Enterprise Edition 2 device, manufactured by Google X Labs, as shown in Figure 4, focusing on visual representations of the results, along with a tablet and a smartphone. Still, any of these devices are valid to use the application.

As illustrated in Figure 4, the Google Glass Enterprise Edition 2 device was used in the experimental tests. Table 1 provides a detailed view of the technical specifications of this device, which are fundamental to the performance and precision of gesture recognition, especially with respect to data processing and the quality and resolution of the camera, which are important for capturing the clear and precise images necessary for the processing of gestures.

### 4.2. Web Application

This system component, accessible on any camera-equipped device with a web browser, consists of a website that hosts the camera feed, overlaid with AR data concerning the lights’ brightness intensity and the graphical representation of hand-recognition landmarks. The development of this page involved the use of Hypertext Markup Language (HTML) and JavaScript (JS). HTML played a crucial role in structuring the overall page layout and design, while JS took charge of orchestrating the AR content through the A-Frame framework, the gesture recognition model from the MediaPipe framework, and the AR.js library.

The A-Frame is an Entity–Component–System (ECS) framework designed for bringing 3D scenes through the web. The A-Frame source code was developed in JS language, making it possible to integrate it with other frameworks and libraries, besides having great compatibility with web browsers since the HTML5 updates. This framework offers several advantages, enabling the web service to maintain a relatively low resource footprint, thus ensuring accessibility across a broad spectrum of mobile devices. This is especially noteworthy, considering the intricacies typically associated with computer graphics technologies.

The MediaPipe framework offers a suite of libraries and tools for applying artificial intelligence (AI) and machine learning (ML) techniques in various applications. These solutions can be customized and utilized on multiple development platforms. The Gesture Recognizer module from MediaPipe was utilized to recognize hand gestures in real-time. It provides the recognized hand gesture results and the hand landmarks of the detected hands.

Eight gestures were employed to control the system, using the Gesture Recognizer module, which already identifies one or two fingers raised, open, and closed. In addition, custom functions were implemented to recognize three or four raised fingers, a pinch gesture, and a raised little finger.

The AR.js library complements the system’s necessary toolbox, providing the functions responsible for recognizing the ArUco tag in the light panel. The ArUco tag makes it possible to identify the board and provides a reference point for superimposing the AR elements on the real-world image captured by the camera.

### 4.3. Middle Server

The middle server plays a pivotal role by hosting the content of the web page, JavaScript libraries, and a middleware function responsible for data exchange with the light panel. To achieve this, the Flask framework was employed. Flask integrates back-end Python programming with front-end HTML and/or JavaScript development, enabling the creation of a full-stack application.

**Figure 5:**
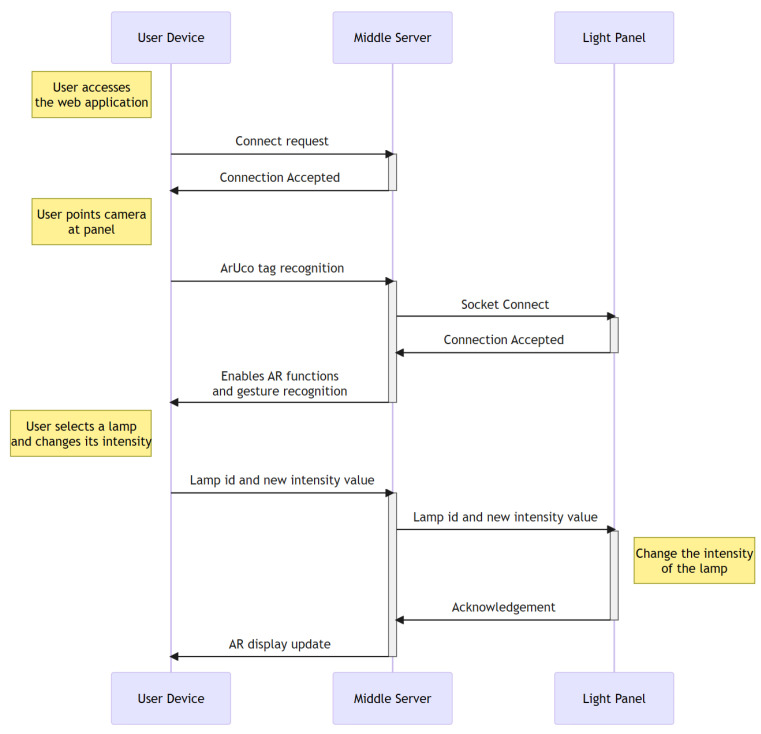
Application time diagram.

Therefore, Flask is used to build a communication bridge, the middle server. It offers both the front-end application accessible by user devices and the back-end application accessible by the panel, facilitating communication. Figure 5 illustrates the communication between the system components, with the middle server acting as an intermediary.

### 4.4. Light Panel

**Figure 6:**
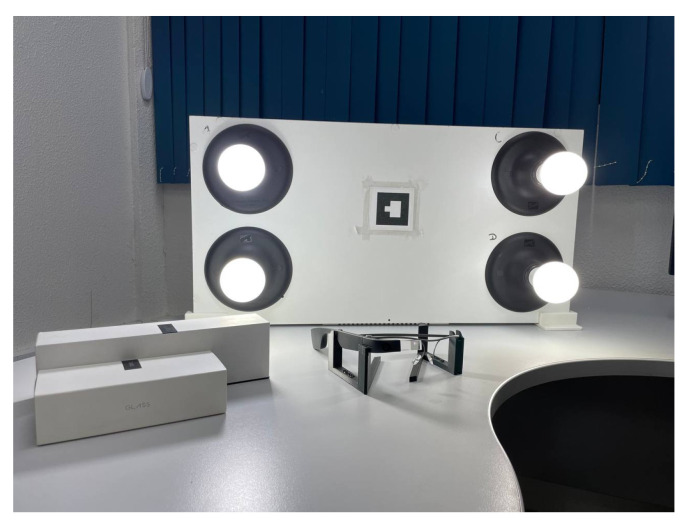
Light panel and Google Glass EE2.

The light panel (Figure 6) comprises a microcontroller, a power control system, and four lamps. The microcontroller serves as the central component of the panel and is responsible for communication with the system, processing incoming data, and generating signals to control the intensity of the lights. The ESP8266 was used as the microcontroller due to its Wi-Fi capability, enabling communication with the middle server over the internet or in a local network.

The power control system consists of a switched mode power supply that converts the 220 V AC input to a 12 V DC output. Subsequently, this output is distributed to two H bridges (L298N). Each H-bridge is equipped with two channels, enabling the powering of two lamps.

The ESP8266 generates four pulse width modulation (PWM) signals based on the intensity values transmitted by the middle server, ranging from 0 to 100%. These control signals are then relayed to each channel of the H-bridges, causing them to replicate the modulation of the received signal over the 12 V power supply, which subsequently regulates the intensity of the lights. This approach allows independent control of the lamps and can be scalable for additional lamps while adhering to the power supply limits.

Finally, the panel has an ArUco tag attached to its front, allowing it to be recognized by the web application.

## 5. Experiments and Discussion

### 5.1. Experimental Tests

**Figure 7:**
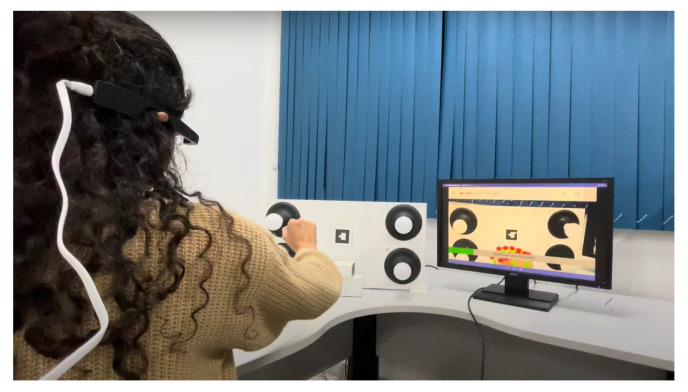
Neutral state.

**Figure 8:**
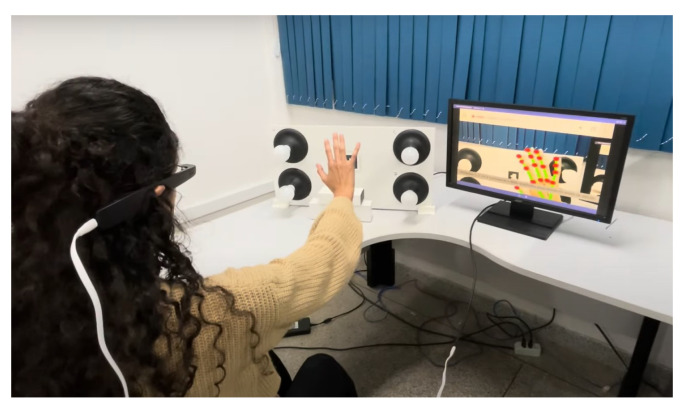
Lamp selection.

**Figure 9:**
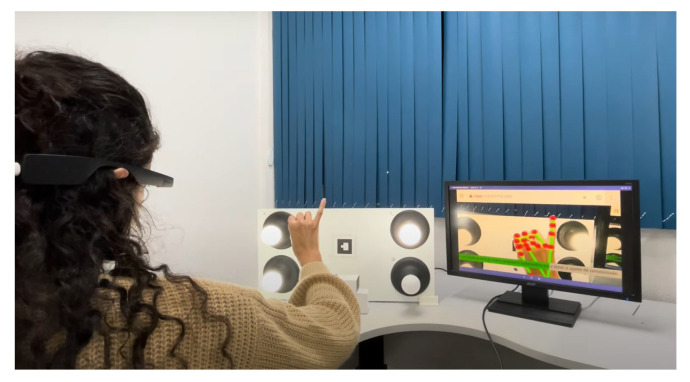
Edition mode.

**Figure 10:**
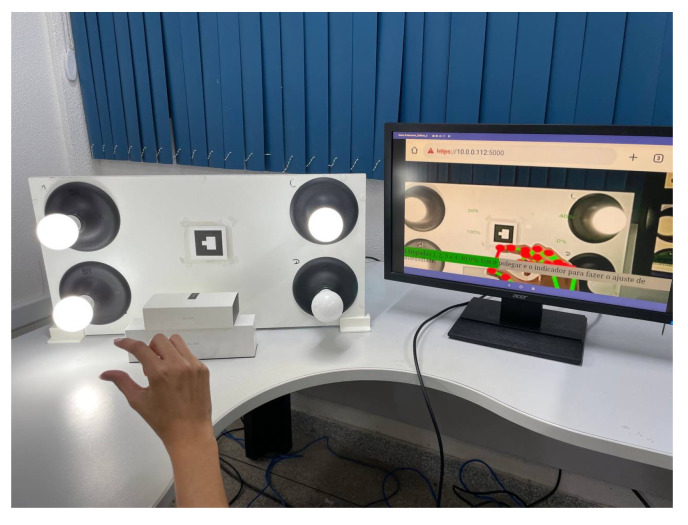
Adjusting lamp brightness.

**Figure 11:**
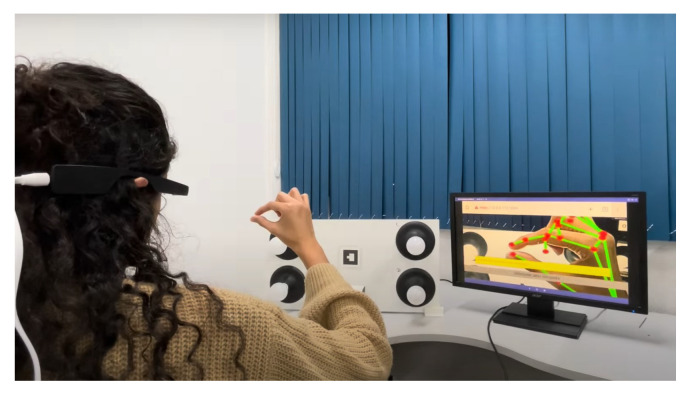
Confirming the selected lamp intensity.

**Figure 12:**
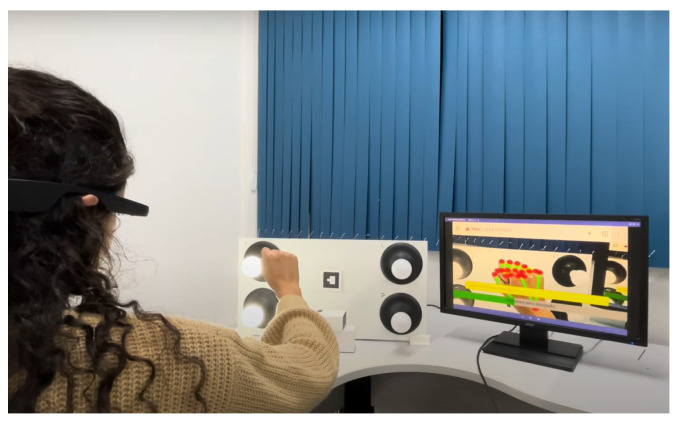
Cancel and return to the previous step.

Focusing on the aspect of user experience, the exploration into enhancing user interaction with technology led this study to conduct tests using a panel of lamps. The characteristic of these lamps is their ability to adjust intensity through a single dynamic hand gesture. The operation of each experiment stage is detailed as follows:

- **Neutral state**
  The process begins with initiating the lamp intensity adjustment. Users are advised to start the control process with a closed hand (Figure 7), representing the neutral state of the system. This initial gesture was chosen for its intuitiveness and ease of use.
- **Lamp selection**
  In the initial phase of adjusting lamp intensity, users are presented with options to select the lamp they wish to modify. This selection can be made in two ways: individually adjusting each light, where the user raises a corresponding number of fingers (1, 2, 3, or 4) or by modifying all lights simultaneously through an open palm gesture (Figure 8). Feedback received from users highlighted the simplicity and efficiency of this selection process, which was refined through a series of design improvements based on an iterative process.
- **Entering editing mode**
  In order to prevent unintended changes and guarantee that the user has complete control over the editing process, the application enters a standby mode. The only way to initiate action during this period is by raising the little finger, which effectively serves as a confirmation gesture (Figure 9). Once the little finger is raised, it directs the user to access the panel’s luminosity intensity parameter editing mode, ensuring that any changes are intentional and avoiding unintentional modifications to the lamp settings.
- **Editing mode: Adjusting the lamp brightness**
  Upon entering the editing mode, users will be prompted to select their desired intensity for the lamp. Adjustment is designed to be carried out using a dynamic pinch gesture, utilizing the proximity of the thumb and index finger to modify the intensity (Figure 10). The closer the fingers are to each other, the lower the intensity value becomes; conversely, the farther apart they are, the higher the intensity value. This change is reflected in real time within the user’s view through a progress bar and an intensity value ranging from 0% to 100%, with intervals of 20%. This approach simplifies the process and enhances the user experience, providing precise and intuitive control of the lamp’s intensity.
- **Editing mode: Confirming the selected lamp intensity**
  An additional step has been introduced to prevent accidental adjustments. To confirm the desired intensity level, the user must rotate their hand to create a 90° angle with the x-axis using the thumb and index finger. In order to help the user, a line is inserted into the hand representation, which turns green when correctly aligned, allowing a 4° margin of error in any direction. Furthermore, the user must maintain the same value while holding the correct angle for 2 s to confirm the selection (Figure 11). If the user alters the value during this period, the timer resets.
  This extra confirmation step ensures that changes to the lamp intensity are deliberate, reducing the risk of accidental adjustments. It also provides a visual and temporal cue to capture user intentions accurately before finalizing the adjustment.
- **Cancel and return to the previous step**
  If the user wishes to cancel an adjustment or change the selected lamp while on the editing screen, they can do so by keeping their hand closed for 2 s (Figure 12).
  This simple and intuitive gesture allows users to easily backtrack or cancel their current action without the need for complex commands. It enhances the system’s overall usability, allowing users to navigate between different stages of the lamp intensity adjustment process easily.
- **Message transmission**
  Once the user confirms the desired change in lamp intensity, the web application generates and sends a message containing the intensity information to the middle server. The middle server then relays this message to the light panel. Upon receiving the message, the light panel adjusts the control signal accordingly, applying the received intensity value to the selected light source.

A video with the complete application usage flow is available on YouTube, through the following link: https://www.youtube.com/playlist?list=PLj2Vk5Dq4cWKYfSbht4NoI8w10hnotTOj (accessed on 10 March 2024) (Real-time Gestural Recognition Playlist).

### 5.2. Discussion

The system’s development incorporated specific techniques, such as normalization, clamping, conversion, and exponential moving average (EMA) filtering, to address technical challenges in dynamic gesture recognition. These techniques were selected based on their ability to handle variations in input data and improve the accuracy of gesture recognition under different usage conditions.

Normalization adjusts the input data to a common scale, facilitating consistent gesture processing, regardless of variations, such as the user’s distance from the device. This step is important to maintain uniformity in the interpretation of gestures captured by the device, which may vary according to the user’s positioning.

Clamping limits the input values within a predetermined range, reducing the impact of extreme values that can lead to inaccurate interpretations. This method is utilized to filter out data that fall outside the expected gesture boundaries, thereby ensuring that the values remain within acceptable bounds.

In addition to the mentioned techniques, converting the Euclidean distance between the thumb and index finger into a percentage value (0% to 100%) is achieved through a flexible method that allows adjustment of minimum and maximum values, representing the physical limits of the pinch gesture. This process is adaptable to work with different ranges of values and measurement units by determining the minimum and maximum clamping values.

Finally, the application of an exponential moving average filter smoothes the data, dampening fluctuations that could be misinterpreted as gestures. This filter is important to filter the noise in the input data, allowing a clearer interpretation of intentional gestures.

The selection of these techniques was motivated by the need to efficiently process captured gesture data in real time, considering the specificities of the hardware used and variable operating conditions. Implementing these approaches contributes to a more reliable system capable of recognizing gestures under different conditions.

- **Normalization**
  The normalization phase of the input data is critical to enhance the accuracy of the gesture recognition system. During the lamp intensity adjustment tests, we noted the need to adjust the data in response to the variations in distance between the hand and the camera. To address these variations, we adopted a normalization process that dynamically modifies the input data to reflect these spatial changes.
  We employed the following formulas, as shown in Equations (1)–(3), to normalize the coordinates of the reference points (*landmarks*) detected on the hand:
  (1) $$distance_{1}=\sqrt{{\left(x_{2}-x_{1}\right)}^{2}+{\left(y_{2}-y_{1}\right)}^{2}+{\left(z_{2}-z_{1}\right)}^{2}}$$
  (2) $$distance_{2}=\sqrt{{\left(x_{4}-x_{3}\right)}^{2}+{\left(y_{4}-y_{3}\right)}^{2}+{\left(z_{4}-z_{3}\right)}^{2}}$$
  (3) $$d=\frac{distance_{2}}{distance_{1}}$$
  In this context, the landmarks are specific points on the hand, represented by red circles in Figure 1 and Figure 2, which were identified through computational vision and play a significant role in determining the position and movement of the hands. The variable *d*, resulting from the distance relationship, serves to adjust the proximity measurement between the thumb and the index finger, facilitating the recognition of the pinch gesture.
  This approach to normalizing the data ensures that the input is consistently adjusted, regardless of the variation in the distance of the hand from the camera or other mutable conditions. This method improves the system’s interpretation of gestures and expands its ability to operate efficiently under a diversity of usage conditions, respecting the specificities of the employed hardware.
- **Clamping**
  Another crucial calculation involved applying a “clamp”, a technique used to limit values within a specified range. As people have variations in the size of their hands and other factors that can influence the results, a clamp becomes important to maintain control. To implement the clamp, we initially defined a range of values. Any value above this defined upper limit was adjusted to 100%, while any value below the specified lower threshold was adjusted to 0%. This clamp operation played a pivotal role in managing individual differences and guaranteeing consistent and meaningful results. It acted as a safeguard, ensuring that values remained within a predetermined and useful range, contributing to the system’s reliability and accuracy.
- **Conversion**
  In the conversion process, we implemented a method to map the Euclidean distance between the thumb and index finger into a percentage scale of 0% to 100%. This mapping is accomplished by defining minimum and maximum values, which represent the physical limits of the pinch gesture, thus allowing considerable flexibility in adjusting these limits for other value ranges and different units of measurement. Determining the minimum and maximum clamping value is important to ensure proper conversion. The following Equations (4)–(7) illustrate the calculation process employed.
  (4) $$n\_intervals=\frac{\left(max\_value-min\_value\right)}{step}$$
  (5) $$range=\frac{\left(max\_clamp-min\_clamp\right)}{n\_intervals}$$
  (6) $$index=\frac{\left(val-min\_clamp\right)}{range}$$
  (7) res=min_value+(step·index)
  These equations facilitate the dynamic mapping of the gesture into a percentage value, considering individual variations in the execution of the gesture. Using this method, the system not only gains in flexibility but also ensures that the conversion of the Euclidean distance into percentage information is performed accurately and consistently, regardless of variations in initial values or the defined step size.
- **Exponential moving average filter**
  It was also observed that as it is a dynamic gesture, having a smaller step (meaning smaller intervals between possible values) and a wider range of possible values made it more challenging to perform the adjustment. This is primarily because the finer granularity of data, resulting from a smaller step, introduces a higher sensitivity to even minor variations in the user’s hand movement. As a user attempts to make precise adjustments, the system encounters more data points and potentially experiences fluctuations in the data due to factors like hand tremors or slight positional shifts. Consequently, these significant data variations can make it more difficult to achieve accurate and stable adjustments in such scenarios.
  To mitigate these challenges, a filtering mechanism was introduced to ensure smoother and more manageable input data, enhancing the overall usability of the system. This filtering mechanism employed an exponential moving average (EMA) approach with a smoothing factor (*f*) set to 0.5.
  For each data point, the formula is as follows.
  (8) v(t+1)=f·v(t)+(1.0−f)·v(t−1)
  This formula calculates the new filtered value (v(t+1)) based on the current data point (v(t)) and the previously filtered value (v(t−1)). The value of the smoothing factor (*f*) was set to 0.5, which means that the new value is a weighted average of 50% of the current data point and 50% of the previous filtered value. This balanced approach helps smooth the data while still responding to changes.
- **Lamp intensity value**
  The intensity of the lamps is recorded as an integer value ranging from 0 to 255, allowing precise control of the PWM signal. This value is converted into a percentage format (0–100%) for user convenience. Information about intensity changes is transmitted between the web application and the middle server and relayed to the light panel as a tuple message ([0, 255]). In this tuple, the first number denotes the lamp index, while the second represents the intensity level. An index with a value of 5 signifies a simultaneous change in intensity for all lamps.

## 6. Conclusions

The framework described in this paper represents progress in the fields of augmented reality and gesture recognition. This project describes the integration and testing of the system with Google Glass. This work encompassed the entire workflow, from selecting the lamps to adjusting their intensity. The results validate the versatility and compatibility of our system and underscore the potential for widespread applications.

In summary, this work represents an advancement in the field of augmented reality and gesture recognition for industrial applications, introducing a web-based architecture that is independent of hardware variations. These distinctive features facilitate a more natural and intuitive interaction with industrial equipment. Implementing this solution proves its technical viability and highlights the potential for improvements in operational efficiency and user experience.

The testing and optimization of our system’s software, including the implementation of techniques such as normalization, clamping, conversion, and filtering, have played a pivotal role in ensuring reliability and accuracy. These technical innovations have standardized hand positions, accommodated individual variations in hand sizes, and allowed precise adjustments. Incorporation of advanced filtering mechanisms has further smoothed user interactions, which is particularly beneficial when handling smaller step sizes and broader value ranges.

This framework has consistently prioritized safety, reliability, and precision, recognizing their paramount importance, particularly in critical real-world contexts such as industrial environments. The design of our system ensures that unintended adjustments or alterations in the physical environment do not occur accidentally, as any unintentional alteration can have significant consequences in critical environments.

This study showed the applicability of a gesture recognition and augmented reality system for industrial applications, offering a perspective on human–machine interaction. The current work has limitations that warrant acknowledgment, including a constrained user interface and the implementation of tests solely within the laboratory setting.

In addition to these efforts, one challenge we aim to address in the future is the issue of the camera recognizing distant hands instead of the intended ones. This can occasionally lead to unintended interactions and should be minimized for an optimal user experience. As part of our ongoing research and development, we will actively work on implementing methods to enhance the system’s hand recognition accuracy, ensuring that it accurately identifies and responds to the user’s gestures, even in scenarios with multiple potential hand sources.

In future works, other algorithms and techniques for gesture recognition and classification can be explored, in addition to investigating performance characteristics based on metrics used in AR systems. Another point of improvement is the possibility of increasing the system’s compatibility with a greater variety of augmented reality devices, in addition to carrying out additional case studies in real industrial environments. Other possibilities are to investigate potential applications beyond the industrial sector, refine the user interface and overall experience, and address specific technical challenges, such as improving remote gesture detection.

## Figures and Tables

**Table 1:** Technical specifications of the Google Glass Enterprise Edition 2.

Specification	Details
Platform	Qualcomm Snapdragon XR1
Display	640 × 360 pixels, RGB
Storage	32 GB
Camera	8-megapixel, 83° diagonal FOV, up to 1080p30 video
Battery life	Up to 8 h
Durability	Lightweight, dust and water-resistant
Device management	Android Enterprise Mobile Device Management
Operating System	Android

## Data Availability

No new data were created or analyzed in this study. Data sharing is not applicable to this article.

## Abbreviations

The following abbreviations are used in this manuscript:

AR	Augmented Reality
VR	Virtual Reality
IMU	Inertial Measurement Unit
JS	JavaScript
HTML	Hypertext Markup Language
ECS	Entity–Component–System
AI	Artificial Intelligence
ML	Machine Learning
AC	Alternating Current
DC	Direct Current
PWM	Pulse Width Modulation
EMA	Exponential Moving Average
